# Prevalence of pathogenic copy number variants among children conceived by donor oocyte

**DOI:** 10.1038/s41598-021-86242-x

**Published:** 2021-03-24

**Authors:** Sandra Monfort, Carmen Orellana, Silvestre Oltra, Mónica Rosello, Alfonso Caro-Llopis, Francisco Martinez

**Affiliations:** 1 Genetics Unit, Hospital Universitario Y Politecnico La Fe, 46026 Valencia, Spain; 2 Department of Genetics, University of Valencia, Valencia, Spain; 3 Genomics Unit, Instituto de Investigacion Sanitaria La Fe, Valencia, Spain

**Keywords:** Genetics, Diseases, Molecular medicine, Risk factors

## Abstract

Development of assisted reproductive technologies to address infertility has favored the birth of many children in the last years. The majority of children born with these treatments are healthy, but some concerns remain on the safety of these medical procedures. We have retrospectively analyzed both the fertilization method and the microarray results in all those children born between 2010 and 2019 with multiple congenital anomalies, developmental delay and/or autistic spectrum disorder (n = 486) referred for array study in our center. This analysis showed a significant excess of pathogenic copy number variants among those patients conceived after in vitro fertilization with donor oocyte with respect to those patients conceived by natural fertilization (p = 0.0001). On the other hand, no significant excess of pathogenic copy number variants was observed among patients born by autologous oocyte in vitro fertilization. Further studies are necessary to confirm these results and in order to identify the factors that may contribute to an increased risk of genomic rearrangements, as well as consider the screening for genomic alterations after oocyte donation in prenatal diagnosis.

## Introduction

Development in the last years of assisted reproductive technology (ART) has favored that nowadays an increasing proportion of the children are conceived with these procedures. Although most children born with these treatments are healthy, some concerns remain regarding the safety of this technology. These concerns are mainly due to the fact that gametes and zygotes in ART treatments are exposed to a series of non-physiological conditions. Some follow-up studies of children conceived by ART suggested that these procedures associated with an increased incidence of epigenetic, genetic or developmental abnormalities^1–5^. On the other hand, some animal model studies support a relationship between ART and an increased risk of diseases such as early-onset diabetes or cardiovascular disease, among others^6,7^. Furthermore, a significant increase of chromosome instability was found in bovine embryos produced by ovum pick up with ovarian stimulation, and in vitro embryos produced from in vitro matured oocytes retrieved without ovarian stimulation in comparison with embryos conceived in vivo^8^. Other studies however did not find an increased risk of birth defects in assisted-conception children compared with naturally conceived children^9,10^.

Nowadays, whole-genome array testing is routinely used in the genetic diagnosis of patients with multiple congenital anomalies or neurodevelopmental disorders, such as intellectual disability or autistic spectrum disorders^11^. In this work, our aim was to examine whether the use of assisted reproduction might imply a higher relative risk of pathogenic copy number variants (CNVs) compared with naturally-conceived children. As far as we know, no similar study has been previously reported in humans. The only similar studies focused on the presence of whole-chromosome aneuploidies in vitrified versus fresh oocytes, where no significant differences were found^12,13^.

## Subjects and methods

A cross-sectional analysis was performed with all those children born in the years 2010 to 2019 submitted for microarray study in our Hospital (n = 486). The reasons for referring included multiple congenital anomalies, developmental delay, intellectual disability and/or autistic spectrum disorder. Among them, 388 were patients conceived by natural fertilization, 5 children by artificial insemination, 34 by autologous oocyte in vitro fertilization (IVF) and 21 children were conceived by donor oocyte IVF. The type of fertilization in 38 patients was not available, and these cases were excluded from further analysis (see Table 1).

**Table1:** Summary of pathogenic CNVs found in patients according to the type of fertilization.

		N	No. pathogenic CNVs	% pathogenic CNVs
Natural fertilization		388	38	10
Assisted reproductive technology				
Artificial insemination		5	0	0
In vitro fertilization	Autologous oocyte	34	4	12
	Donor oocyte	21	9	43
Unknown		38	1	3
Total		486	52	11

The IVF pregnancies by egg donation were performed in different private fertility clinics. It is a common practice in these clinics to perform a genetic screening on all potential donor candidates prior to donation, including studies such as karyotype, cystic fibrosis, fragile-X, spinal muscular atrophy and it is becoming popular the use of different panels for recessive mutations by NGS. The donors must be young, by law under 35, and in several clinics, preferably under 30 years old.

The Affymetrix CytoScan 750 K SNP array were used for all studies, and the manufacturer’s quality recommendations (MAPD < 0.25, snpQC > 12, Waviness-SD < 0.12) were strictly followed. The results were analyzed, prior to the present study, with the Affymetrix Chromosome Analysis Suite v.3.1 software as recommended by the manufacturer. Copy number calls were a priori filtered for > 50 probes, with no limit of size. These pathogenic CNVs were highly reliable, not only for the large number of altered copy-number probes, but because they were independently supported by an altered pattern of the allele difference tracks of SNPs. In addition, a more detailed inspection (above 20 altered probes) was performed in genes implied in developmental disorders, with special relevance of those for which pathogenic de novo deletions and/or loss-of-function mutations have been reported, that is with proved haploinsufficiency. CNV classification was applied following widely approved recommendations reported elsewhere and systematically employed in our center^14,15^. Basically, pathogenic CNVs were classified according to the following criteria: (1) those CNVs previously reported as pathogenic, not reported (or exceptionally) in healthy carriers; (2) CNVs causing the loss or gain of dose-sensitive genes responsible for specific syndromes; (3) those CNVs larger than 2 Mb affecting above 50 coding genes, which do not overlap benign CNVs reported in healthy controls^16^; (4) firmly established pathogenic CNVs with incomplete penetrance, which can be present in healthy carriers, but associate higher predisposition to neurodevelopmental disorders^17^. This study was approved by the Hospital La Fe Ethics Committee and authorizations from all the patients and the participating relatives were obtained by signing an informed consent form. This research was carried out in accordance with the relevant guidelines and regulations.

## Results

Fifty-two patients with pathogenic CNVs were detected in our cohort (detailed in online Supplementary Table S1), all of them born of healthy parents, or supposedly healthy egg or sperm donors. It is important to note that clinical interpretation of these CNVs was carried out previously to the present analysis. Thirteen of these pathogenic CNVs were found in patients conceived by in vitro fertilization, being remarkable that nine of these patients were conceived from donor oocyte. Consequently, the rate of pathogenic CNVs found in the ovodonation group of patients (9 out of 21; 43%) is higher than in the other groups: four by autologous oocyte IVF (4/34; 12%) and 38 by natural fertilization (38/388; 10%) (see Table 1).

Our data indicate a clear excess of pathogenic CNVs in the group of donor oocyte IVF with respect to those patients conceived by natural fertilization, (p = 0.0001; Fisher's exact test). Conversely, among the patients born by autologous IVF, the frequency of pathological CNVs does not differ from that observed in cases conceived by natural fertilization (p = 0.76). A potential bias could due to an increase of the paternal age in conceptions by egg-donation. However, the mean paternal age among these cases does not significantly differ from the general population (35.5 years vs. 34.5 respectively), or from the control group (34.7 years).

On the other hand, we also consider whether in our series there is an excess of patients born through ART, based on epidemiological data obtained from national registers^18,19^. According to the National Registry of the Spanish Fertility Society, during the period 2010–2017 a total of 66,790 newborns were born after autologous oocyte IVF, which represent 1.91% of all newborns in Spain during these years, 37,310 after donor oocyte IVF (1.01%) and 27,568 after artificial insemination (0.79%)^19^. If there was no association, the proportion of children born by oocyte donation among those patients bearing pathogenic CNVs would be expected to be similar to that of the general population, i.e. about 1–2%, however we found that this proportion is much higher than expected (9/52 = 17.3%; 95% CI = 9.4–29.7%). By comparison, the ratio of patients with pathogenic CNVs who were conceived by other assisted reproduction techniques (4/52 = 7.7%; 95% CI = 3.0–18.2%) is only slightly increased with the proportion expected from this same registry (2.7% including autologous oocyte IVF and artificial insemination).

**Figure 1:**
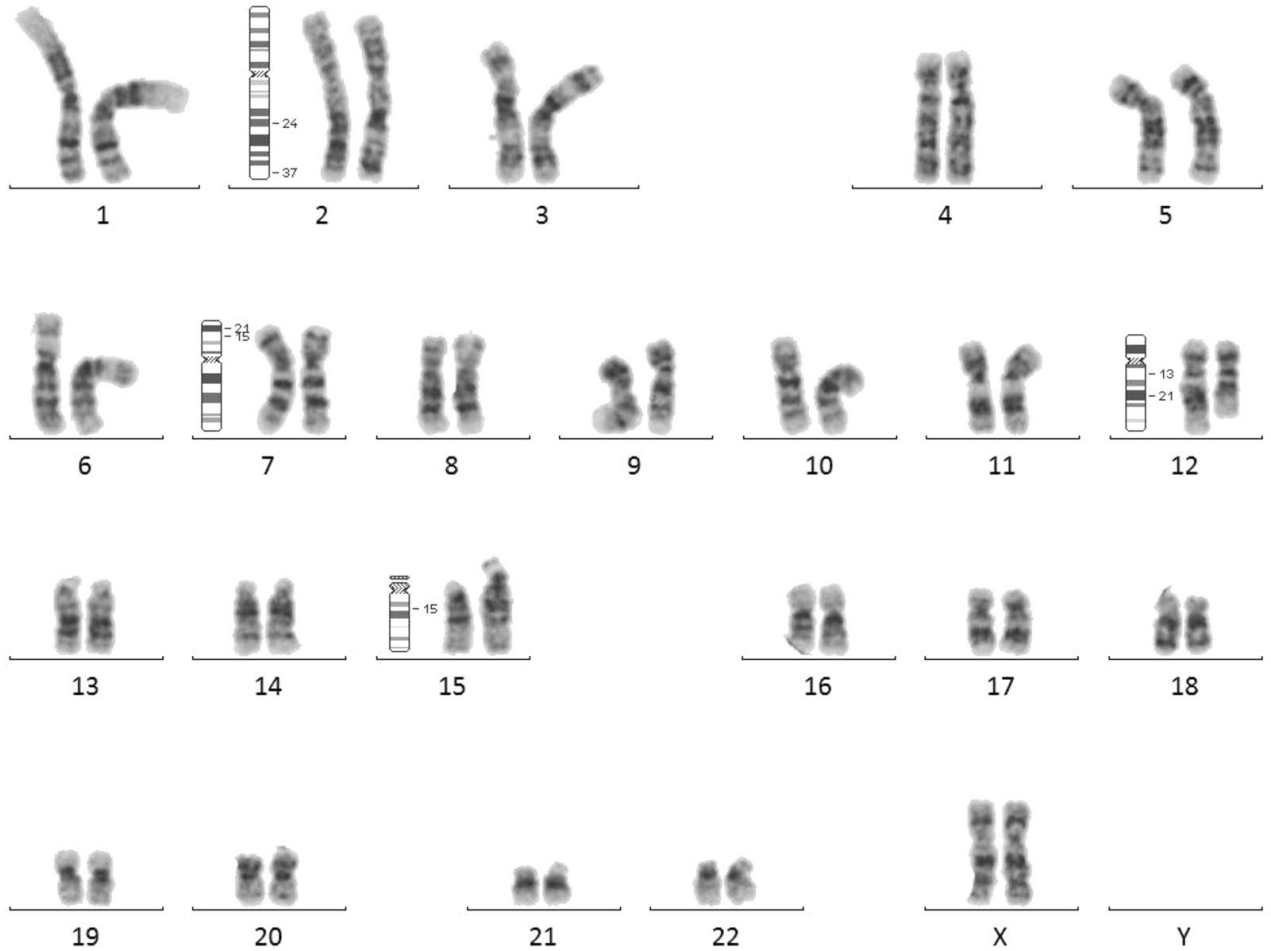
Karyotype of case 12 was 46,XX,inv(2)(q24q37),del(7)(p21.3p15.3)ins(15;12)(q15;q13q21), the patient presents a complex rearrangement with a partial deletion of the short arm in chromosome 7 with breakpoints in bands p13.3 and p15.3, that is in the limit of resolution of cytogenetic techniques, previously detected by genomic array. Also a chromosome 2 with a paracentric inversion in the long arm with breakpoints in the bands q24 and q37 and a smaller chromosome 12 with part of long arm material inserted into chromosome 15.

**Figure 2:**
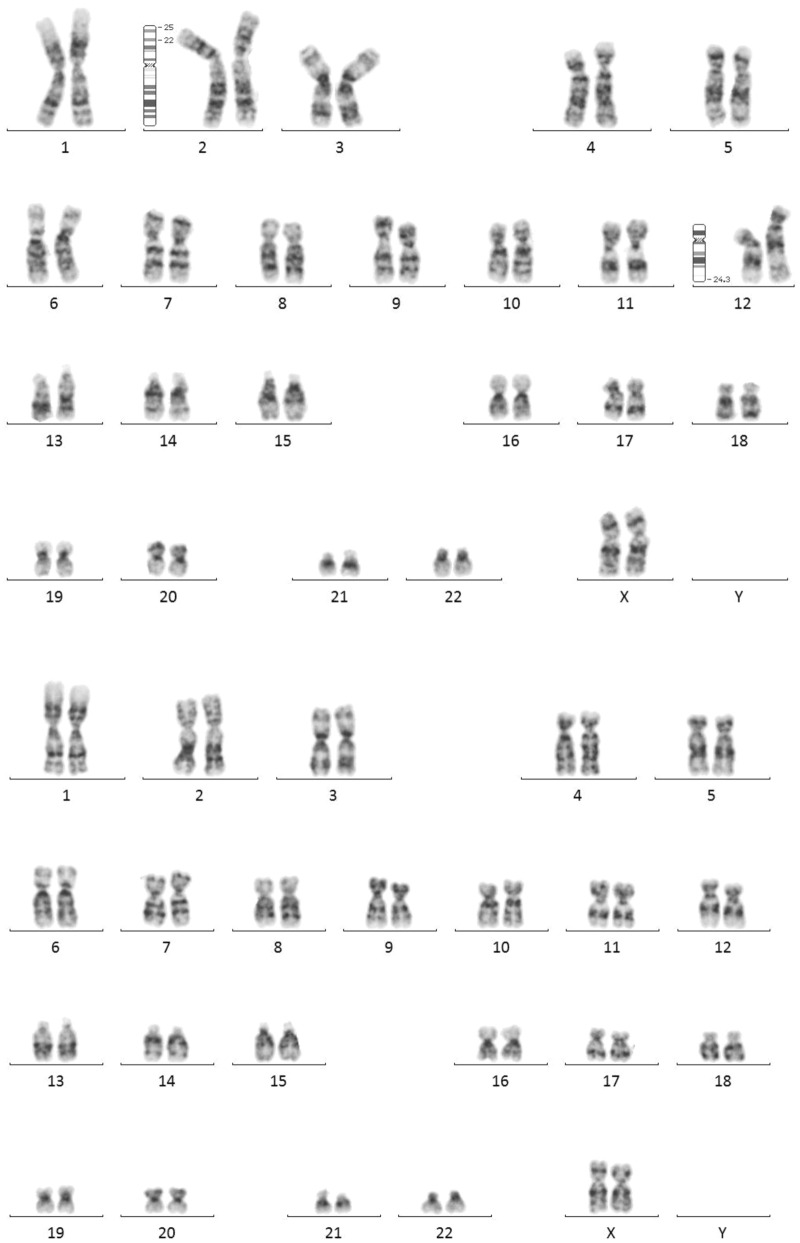
Karyotype of case 13 was 46,XX,der(12)ins(12;2)(q24.3;p22.1p25.3)[33]/46,XX[20] The patient presents a duplication of the short arm of chromosome 2 with breakpoints in bands p22.1 and p25.3 The duplication region was inserted in the long arm of chromosome 12 in band q24.3 in 60% of cells. A similar proportion was obtained by FISH analysis on oral mucosa cells (not shown).

**Figure 3:**
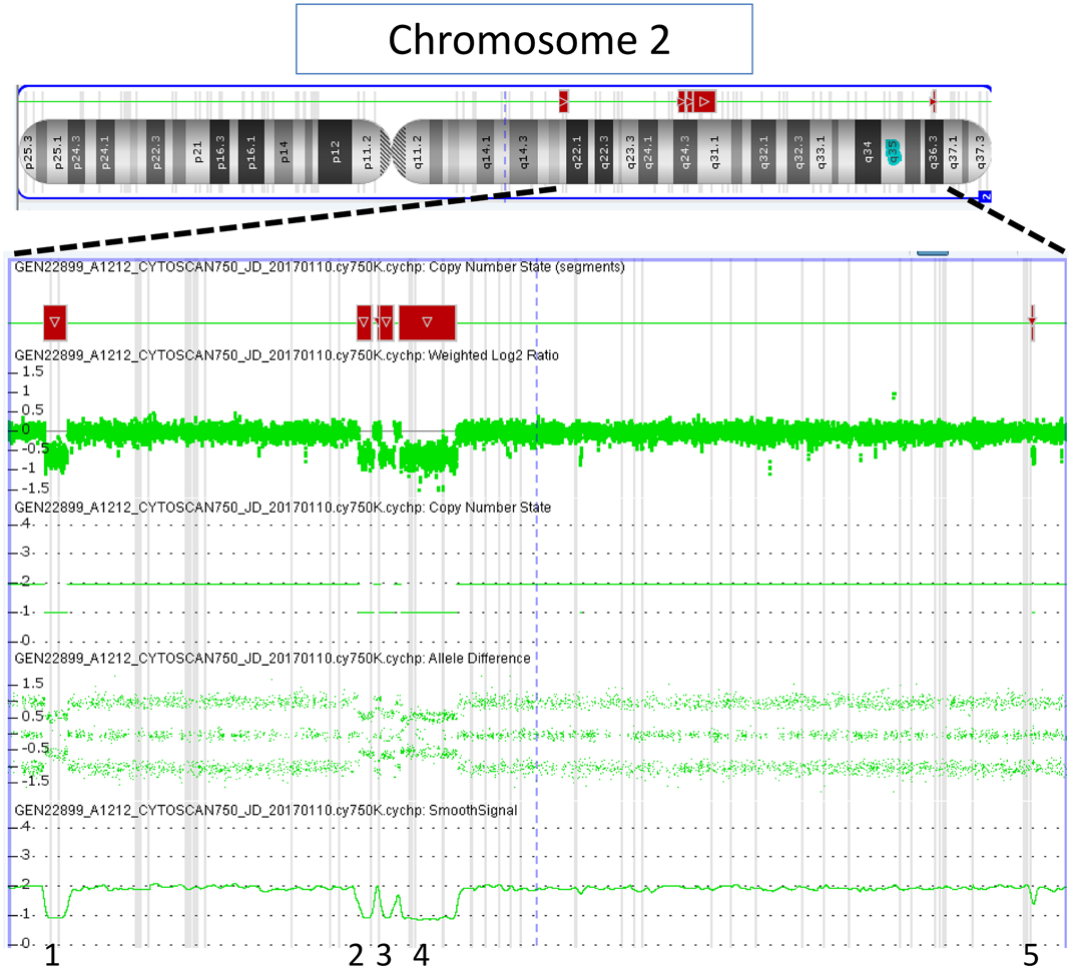
Result of the Affymetrix CytoScan 750 SNP array in patient 15: In the upper part, ideogram of chromosome 2 with the deleted regions marked with black boxes. In the lower part, detailed view the long arm of chromosome 2 where the different deletions are visualized.

Furthermore, it should be noted that among the cases with pathological CNVs born after oocyte donation, two of them presented very complex chromosomal rearrangements that are exceptionally observed in routine cytogenetic studies (Figs. 1 and 2). Another patient conceived by means of IVF techniques after vitrification of autologous gametes, also has a complex rearrangement consisting of 5 deletions of the long arm of chromosome 2 (Fig. 3).

## Discussion

We have detected a putative association between the donor oocyte IVF and the presence of pathogenic CNVs in our series of patients born with multiple congenital anomalies, developmental delay and/or autistic spectrum disorder submitted for microarray study during the years 2010–2019 (p = 0.0001). It can be assumed that the results of the genetic screening for oocyte donors were negative, and that the possibility that oocyte donors were carriers of rare pathogenic CNVs is unlikely. Conversely, among patients born by autologous oocyte IVF, the ratio of pathogenic CNVs is very similar to those cases born by natural fertilization (p = 0.76). Consequently, our results confirm (and complement) a recently published study, based on population registers in Western Australia, where an increased risk of intellectual disability was found in the children conceived by ART, even after stratification for twin pregnancies or very preterm births^1^. Significantly, these authors found that patients conceived by ART were more likely to have a known genetic cause than naturally conceived patients (20% vs. 12%), which is in accordance with our results.

It is also worth noting that among the cases with pathological CNVs born after oocyte donation, two of them presented exceptionally complex chromosomal rearrangements. Patient 12 showed a deletion of 7.9 Mb of chromosome 7, and the karyotype subsequently showed a complex rearrangement involving four chromosomes and seven breakpoints (Fig. 1). In patient 13, a mosaicism for a 39 Mb duplication of chromosome 2 was confirmed by peripheral blood karyotype, with a 62% of the cells showing a complex rearrangement (Fig. 2). The high-degree mosaic status was confirmed by fluorescent in situ hybridization (FISH) study on oral mucosa. Given the high degree of mosaicism found in cells of different embryonal origin, the complex rearrangement must have occurred in the early stages of the embryonic development. On the other hand, patient 23, conceived by IVF techniques with vitrified autologous gametes, showed a complex rearrangement with 5 different deletions on chromosome 2 (Fig. 3), probably caused by a chromothripsis phenomenon. These rearrangements are very rare catastrophic events that occur during a cell division cycle that originate multiple losses and/or gains of chromosomal material^20^.

We do not know the mechanism that could be favoring the association. One possibility could be that some of the procedures employed for oocyte donation would favor, at least in some individuals with special conditions of susceptibility, a defective repair system of the DNA. The mature oocyte is well provided with a fully functional DNA repair system that takes care of both the maternal and the paternal genomes during the cleavage divisions after fertilization. Vitrification of oocytes is usually applied in the majority of assisted reproduction clinics for the oocyte donation process. During this procedure, the oocyte is exposed to physical and chemical processes under non-physiological conditions which might affect the integrity of the genome or its ability to repair^21^. In this sense, oocyte vitrification procedures involve the use of products that could directly or indirectly favor the appearance of genomic rearrangements or affect DNA repair capacity. Currently the information available on the impact of cryopreservation/vitrification of oocytes on the genome is rather scarce. Some studies have evaluated the percentage of aneuploidies in vitrified versus fresh oocyte of the same couple, and no significant differences were found^12,13^. However, as far as we know, no published work has been so far focused on the putative effect of vitrification or donor oocyte on a higher rate of intra-chromosomal deletions or duplications (CNVs) in humans. A study on single blastomeres from bovine embryos found that the frequency of aneuploidies or segmental rearrangements was higher in embryos produced in vitro (with or without ovarian stimulation) than in embryos derived in vivo, although their data are rather preliminary^8^.

Our results suggest a higher frequency of pathogenic copy number variations in children conceived after oocyte donation. If this higher risk in fact exists, the procedures performed in these techniques of assisted reproduction should be reviewed in order to identify the factors that may contribute to this increased risk of genomic rearrangements. In addition, a prenatal diagnosis should be considered in those pregnancies in order to detect any possible pathogenic CNV.

## Supplementary Information

Supplementary Information

## Data Availability

The data that supports the findings of this study are available in the supplementary material of this article.
